# Pathobiology and Therapeutic Relevance of GSK-3 in Chronic Hematological Malignancies

**DOI:** 10.3390/cells11111812

**Published:** 2022-05-31

**Authors:** Alberto M. Martelli, Francesca Paganelli, Camilla Evangelisti, Francesca Chiarini, James A. McCubrey

**Affiliations:** 1 Department of Biomedical and Neuromotor Sciences, University of Bologna, 40126 Bologna, Italy; francesca.paganell16@unibo.it (F.P.); camilla.evangelisti@unibo.it (C.E.); 2 CNR-Institute of Molecular Genetics “Luigi Luca Cavalli-Sforza”, Unit of Bologna, 40136 Bologna, Italy; francesca.chiarini@cnr.it; 3 IRCCS Istituto Ortopedico Rizzoli, 40136 Bologna, Italy; 4 Department of Microbiology and Immunology, East Carolina University, Greenville, NC 27834, USA; mccubreyj@ecu.edu

**Keywords:** GSK-3, paralogs, chronic hematological malignancies, targeted therapy, chronic myelogenous leukemia, chronic lymphocytic leukemia, multiple myeloma, B-cell non-Hodgkin’s lymphomas

## Abstract

Glycogen synthase kinase-3 (GSK-3) is an evolutionarily conserved, ubiquitously expressed, multifunctional serine/threonine protein kinase involved in the regulation of a variety of physiological processes. GSK-3 comprises two isoforms (α and β) which were originally discovered in 1980 as enzymes involved in glucose metabolism via inhibitory phosphorylation of glycogen synthase. Differently from other proteins kinases, GSK-3 isoforms are constitutively active in resting cells, and their modulation mainly involves inhibition through upstream regulatory networks. In the early 1990s, GSK-3 isoforms were implicated as key players in cancer cell pathobiology. Active GSK-3 facilitates the destruction of multiple oncogenic proteins which include β-catenin and Master regulator of cell cycle entry and proliferative metabolism (c-Myc). Therefore, GSK-3 was initially considered to be a tumor suppressor. Consistently, GSK-3 is often inactivated in cancer cells through dysregulated upstream signaling pathways. However, over the past 10–15 years, a growing number of studies highlighted that in some cancer settings GSK-3 isoforms inhibit tumor suppressing pathways and therefore act as tumor promoters. In this article, we will discuss the multiple and often enigmatic roles played by GSK-3 isoforms in some chronic hematological malignancies (chronic myelogenous leukemia, chronic lymphocytic leukemia, multiple myeloma, and B-cell non-Hodgkin’s lymphomas) which are among the most common blood cancer cell types. We will also summarize possible novel strategies targeting GSK-3 for innovative therapies of these disorders.

## 1. Introduction

Chronic malignant hematological disorders comprise a highly heterogeneous group of blood diseases, arising from the neoplastic transformation of either myeloid or lymphoid cells, which are usually characterized by a relatively slow and indolent course. The most common of these disorders include chronic myeloid leukemia (CML), chronic lymphocytic leukemia (CLL), multiple myeloma (MM), and B-cell non-Hodgkin’s lymphomas (B-cell NHLs) [1,2]. Historically, the mainstays of therapy for these diseases have been chemotherapy, radiotherapy, and bone marrow transplantation (BMT) [3,4,5,6].

However, over the last two decades, more effective therapeutic regimens have emerged. Targeted therapy with tyrosine kinase inhibitors (TKIs) has been extremely successful in CML patients, where TKIs have drastically changed the natural history and the outcome of the disease [7]. Targeted therapeutics have proven to also be beneficial in CLL [8] and MM [9]. Immunotherapies (e.g., monoclonal antibodies, bispecific antibodies, immune checkpoint inhibitors, and CAR-T therapy) display remarkable efficacy in some types of B-cell NHLs, CLL, and MM [10,11,12]. Nevertheless, despite striking therapeutic progresses, CLL, MM, and some B-cell NHL subtypes still remain incurable diseases. As to CML, intrinsic or acquired resistance to TKIs is present in several patients and represents a formidable hurdle for achieving a definitive cure [13].

Owing to the aberrant regulation of protein kinase activity in many cancer types, this enzyme family has become one of the most important drug targets in the 21st century. At present, at least 58 therapeutic agents, which target about two dozen different protein kinases, have been approved by the Food and Drug Administration (FDA) for cancer treatment [14]. Although most protein kinase inhibitors have not lived up to their expectations [15], there is still room for reinvestigating kinases as possible targets for improving the therapy of incurable blood cancers. 

Glycogen synthase kinase-3 (GSK-3) is a serine/threonine protein kinase which was isolated over 40 years ago in rabbit muscle and was so named based on its ability to phosphorylate and inhibit glycogen synthase, a key negative regulator of glycogen synthesis [16]. GSK-3 is an ancient and conserved broad specificity kinase, expressed in most of the vertebrates, which critically controls several signaling networks; hence, it is involved in a variety of physiological functions, including embryonic development, sperm maturation, apoptosis, autophagy, metabolism, neurogenesis, and synaptic plasticity [17,18,19,20,21]. 

Over the last three decades, GSK-3 has also emerged as a kinase clearly implicated in the pathogenesis and progression of a wide spectrum of human disorders, including cardiovascular diseases, type 2 diabetes, chronic inflammation, bipolar disorder, neurodegenerative disorders (e.g., Alzheimer’s disease, Parkinson’s disease, Huntington’s disease), and cancer [22,23,24,25,26,27,28]. As far as cancer is concerned, GSK-3 was initially considered to be a tumor suppressor [29]. Nevertheless, numerous, more recent studies have disclosed a tumor-promoting role for GSK-3 isoforms in some cancer settings [26]. Given the involvement of GSK-3 in a broad range of human diseases, drugs targeting GSK-3 represent emerging tools for clinical intervention, especially in combination with other treatments [26,30]. 

In this review, we summarize our current knowledge of GSK-3 relevance in the pathobiology of some of the most common chronic blood cancers. Furthermore, we highlight how novel therapeutic strategies targeting GSK-3 might be employed for improving the outcome of these malignant disorders.

## 2. An Overview of GSK-3 Signaling

**Figure 1:**
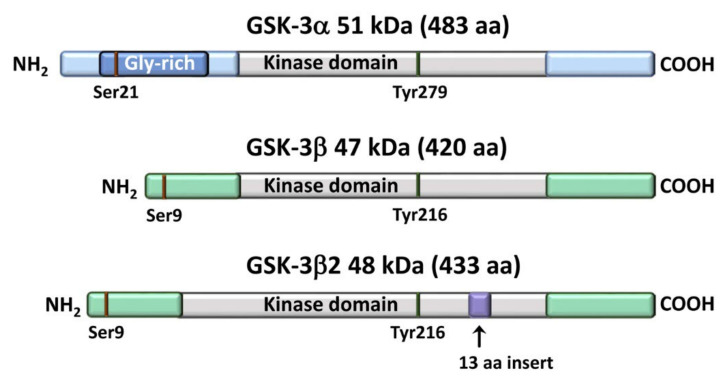
Structural domains and main sites of phosphorylation of GSK-3 isoforms.

In mammals, GSK-3 comprises the α (51 kDa) and β (47 kDa) isoforms or, more precisely, paralogs, because they are homologous proteins encoded by two distinct genes (*GSK3A* and *GSK3B*). In humans, *GSK3A* is located on chromosome 19q13.2, while *GSK3B* is on chromosome 3q13.3 [31]. The GSK-3 paralogs share almost identical (>95% homology) kinase domains but differ considerably at their NH_2_- and COOH-termini. In particular, GSK-3α has a glycine-rich NH_2_-terminal extension, which is missing in GSK-3β. Overall, the isoforms display 84% amino acid sequence homology [32]. There exists a splice variant of GSK-3β (GSK-3β2, 48 kDa) which displays a 13 amino acid insert in the kinase domain [33] (Figure 1). GSK-3β2 is expressed exclusively in the nervous system [34].

GSK-3α and GSK-3β are expressed in all human tissues and organs and share some common substrates [35]; however, they also exhibit specific biological roles. Indeed, despite the high degree of structural homology, the ablation of one isoform could not be fully compensated for by the other, as shown by studies performed in murine models [36,37,38]. Regarding its subcellular localization, GSK-3 is considered to be largely a cytosolic enzyme. However, a pool of GSK-3 continuously shuttles in/out of the nucleus [39,40]. Notably, in some cancer types, an aberrant upregulation of the nuclear GSK-3 pool has been linked to elevated levels of nuclear factor-κB (NF-κB)-dependent gene transcription [41,42]. 

**Figure 2:**
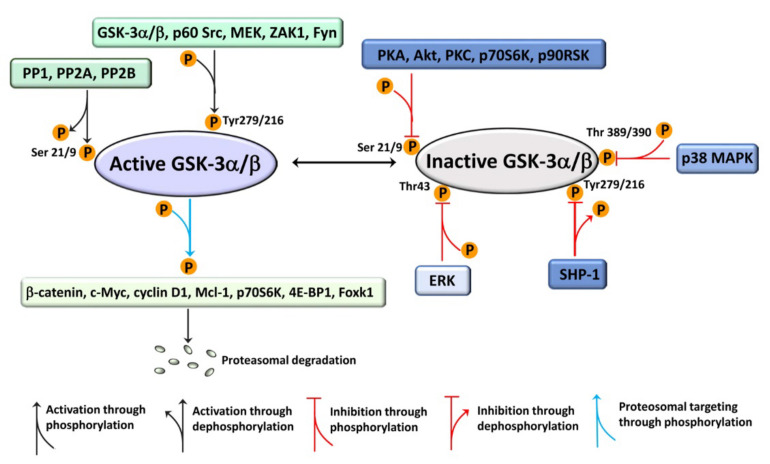
Overview of GSK-3 isoform activation control and downstream signaling. In resting cells, GSK-3 isoforms are constitutively active, i.e., phosphorylated on Tyr 279/216 residues. These residues are targeted by GSK-3 itself as well as by other kinases (p60 Src, MEK, ZAK1, Fyn). Phosphotyrosines are dephosphorylated by the protein phosphatase SHP-1. Upon a variety of external cues, phosphorylation on Ser21 (GSK-3α) or Ser9 (GSK-3β) downregulates the enzymatic activity. Serine residues are targeted by a variety of upstream kinases (PKA, Akt, PKC, p70S6K, and p90RSK). Phosphorylation on Ser 21/9 residues is facilitated by phosphorylation on Thr43 by ERK and on Thr 389/390 by p38 MAPK. The protein phosphatases PP1, PP2A, and PP2B dephosphorylate the serine residues, thereby activating GSK-3 paralogs. Active GSK-3 isoforms phosphorylate a broad range of substrates (β-catenin, c-Myc, cyclin D1, Mcl-1, p70S6K, 4E-BP1, Foxk1, etc.) which are then proteolytically degraded via the proteasome.

Differently from most protein kinases, GSK-3 isoforms are constitutively active in resting cells, partly due to autophosphorylation of GSK-3α on Tyr279 and of GSK-3β on Tyr216. These phosphorylations, which take place during GSK-3 translation, upregulate the enzymatic function fivefold, as they could facilitate substrate binding [43,44]. Nevertheless, kinases capable of phosphorylating the GSK-3 isoforms on the Tyr279/216 residues have been discovered. These include mitogen-activated protein kinase (MEK) [45], the nonreceptor tyrosine kinase p60 Sarcoma kinase (p60-Src), Zaphod kinase 1 (ZAK1), and Fyn [44]. In contrast, extracellular cues such as hormones (e.g., insulin), growth factors (e.g., epidermal growth factor (EGF), platelet-derived growth factor (PDGF)), neurotransmitters, or reactive oxygen species (ROS) initiate intracellular signaling pathways which lead to phosphorylation of GSK-3α on Ser21 and of GSK-3β on Ser9, thereby inactivating (although not completely) GSK-3 isoforms [46]. Multiple kinases phosphorylate the serine residues, including protein kinase A (PKA), protein kinase B (PKB or Akt), protein kinase C (PKC), the mechanistic target of rapamycin (mTOR), p70 ribosomal S6 kinase (p70S6K), and p90 ribosomal S6 kinase (p90RSK) [47,48]. This is important as it allows several signaling pathways to converge on GSK-3 which then acts as an integrator of signals, thereby allowing normal cells to maintain homeostasis. Furthermore, phosphorylation on either Thr43 by extracellular signal-regulated protein kinase (ERK) or on Thr389/390 by p38 mitogen-activated protein kinase (p38 MAPK) inhibit GSK-3, as these events facilitate subsequent phosphorylation on the Ser 21/9 residues [49,50] (Figure 2). Phosphatases counterbalance the effects of kinases on GSK-3 isoforms. The phosphotyrosine residues are targeted by protein phosphatase Src homology-2 (SH2) domain-containing phosphatase 1 (SHP-1) [51,52], while the serine residues are dephosphorylated by protein phosphatase 1 (PP1), protein phosphatase 2A (PP2A), and protein phosphatase 2B (PP2B) [53,54,55] (Figure 2). 

It is also beginning to emerge that GSK-3β is mutated in approximately 1–4% of several tumor types (e.g., uterine endometrioid carcinoma, skin cancer, uterine neoplasms, melanoma, non-small-cell lung cancer, and cervical squamous cell carcinoma). Several of the mutations identified so far are in the kinase domain. Therefore, some mutations might impact the activity of GSK-3β as either a tumor suppressor or a tumor promoter, depending on the downstream targets and tumor types [56]. Furthermore, microRNAs (miRs) have been shown to regulate GSK-3 expression in some cancer settings [30,57,58].

Over 100 proteins have been reported to be phosphorylated by GSK-3. However, the functional consequences of phosphorylation by GSK-3 have been identified for only about 40 of these substrates. A distinctive and unusual feature of GSK-3 substrates is that they are usually prephosphorylated (primed) by another kinase. In other words, the most common site for phosphorylation by GSK-3 isoforms is a prephosphorylated motif, S/T-X-X-X-S/T(P), where GSK-3 phosphorylates a serine/threonine 4 aminoacidic residue to the NH_2_-terminal side of the GSK-3 priming site [59]. Although substrate priming is not an absolute requirement, there is a 1000-fold increase in phosphorylation efficacy by GSK-3 for primed targets. Priming kinases include ERK, p38 MAPK, c-Jun N-terminal kinase (JNK), and 5′-adenosine monophosphate-activated protein kinase (AMPK). 

Apart from its role in glycogen metabolism, GSK-3 phosphorylates β-catenin [60], cyclin D1 [61], c-Myc [62], myeloid leukemia cell differentiation protein 1 (Mcl-1) [63], tuberous sclerosis 2 (TSC2) [64], rapamycin-insensitive companion of tor (Rictor) [65], regulatory-associated protein of tor (Raptor), and the Snail family of transcription factors [66]. GSK-3 targets p70S6K and eukaryotic translation initiation factor 4E (eIF4E)-binding protein 1 (4E-BP1), two key regulators of protein translation [67,68]. GSK-3 also controls cell metabolism via the forkhead/winged helix family k1 (Foxk1) transcription factor [69], as well as mitochondrial functions through the phosphorylation of proapoptotic B-cell lymphoma 2 (Bcl-2)-associated X (Bax) protein [70] (Figure 2). Therefore, many GSK-3 substrates are involved in the regulation of the hallmarks of neoplastic cells, including sustained proliferation, resistance to apoptosis, deregulated metabolism, invasion and metastasis, angiogenesis, genome instability, etc. [71].

Typically, proteins phosphorylated by GSK-3 are recognized by E3 ubiquitin ligases and targeted for degradation via the proteasome [72] (Figure 2). As a consequence, GSK-3 is a negative regulator of signaling pathways which are crucial for cancer cells’ proliferation and survival, such as wingless (Wnt)/β-catenin, Sonic Hedgehog (SHH), Neurogenic locus notch homolog protein (Notch), growth factor/tyrosine kinase receptor (TKR), and G-protein-coupled receptor signaling networks [73].

**Figure 3:**
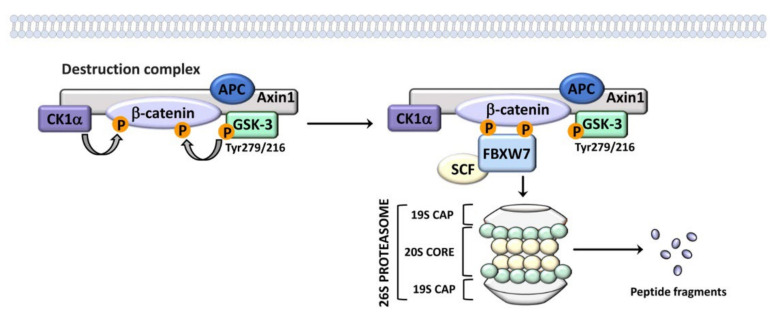
Active GSK-3 isoforms are components of the “destruction complex” which targets β-catenin to degradation through the proteasome. The “destruction complex” also includes Axin1, APC, CK1α, and β-catenin. CK1α targets β-catenin on the Ser45 residue, thereby priming β-catenin for phosphorylation by GSK-3α/β on multiple aminoacidic residues (Ser33/37, Thr41). Phosphorylated β-catenin is recognized by the FBXW7/SCF complex and degraded through the proteasome.

It is important to emphasize that GSK-3 isoforms act as negative regulators of the canonical Wnt/β-catenin network via the degradation of cytoplasmic β-catenin [26]. GSK-3 is a component of the multiprotein destruction complex which includes Axin inhibition protein 1 (Axin1), the tumor suppressor adenomatous polyposis coli (APC), casein kinase 1α (CK1α), and β-catenin. CK1α targets β-catenin on the Ser45 residue, thereby priming it for phosphorylation by GSK-3 on Ser33/37 and Thr41 amino acidic residues. Since GSK-3 does not directly bind β-catenin, Axin1 and APC mediate the interactions of β-catenin with GSK-3 [74]. Phosphorylated β-catenin is recognized by the F-box/WD repeat-containing protein 7 (FBXW7)/S-phase kinase-associated protein 1, Cullin, and F-box containing complex (SCF), and degraded via the proteasome [75] (Figure 3). Thus, GSK-3 suppresses the Wnt/β-catenin signaling axis [18,76] which is often overactive in cancer, including several types of blood malignancies [77]. Interestingly, the effects of GSK-3 isoforms on Wnt signaling are independent from phosphorylation on Ser 21/9 [78,79]. Wnt likely inhibits GSK-3 by a still-poorly defined mechanism which is thought to involve disruption of the degradation complex [80]. 

When Wnt signaling is not inhibited, β-catenin migrates to the nucleus, where it upregulates specific transcriptional programs leading to the expression of proto-oncogenes such as cyclin D1, c-Myc, vascular endothelial growth factor (VEGF), and matrix metalloproteinase-7 (MMP-7) [81]. 

Since the genetic loss of either GSK-3 paralog does not lead to the accumulation of β-catenin in the cytoplasm of murine embryonic stem cells, the two isoforms were considered redundant, at least from this point of view [79]. Nevertheless, subsequent studies indicate that the GSK-3 paralogs could play context-dependent, distinct roles in the control of β-catenin proteolysis [82]. 

### 2.1. GSK-3 Signaling in Healthy B-Cells

CLL, MM, and B-cell NHLs are B lineage-derived neoplastic disorders. Therefore, it seems important to briefly summarize our current knowledge of the roles played by GSK-3 isoforms in healthy B-cells. Early studies demonstrated that GSK-3β negatively regulates Wnt/β-catenin signaling in murine pro-B-cells, thereby limiting their proliferation and survival [83]. More recent studies have focused on the involvement of GSK-3β in the physiology of germinal center (GC) B-lymphocytes. GCs are transient microstructures located in the center of B-follicles (for example in lymph nodes) where antigen-driven somatic hypermutation occurs. Moreover, GCs are the site of generation of affinity-matured plasma cells and memory B-cells capable of mediating long-term protective immunity in response to signals received by receptors [84]. For the scope of this review, it is also important to emphasize that B-cell receptor (BCR) signaling plays a role of the utmost importance in the survival of GC B-lymphocytes [85].

GSK3-β is phosphorylated on Ser9 (hence inactivated) by PKA in GC B-lymphocytes. As a consequence, cyclin D3 expression levels are higher, thereby driving B-cell proliferation and expansion in GCs after the initial encounter with T-cells [86]. Both genetic ablation and pharmacological inhibition demonstrated that, upon stimulation with CD40L and interleukin (IL) -21, the inactivation of GSK-3β in murine GC B-cells facilitated the formation of plasma cells. Mechanistically, inhibition of GSK3-β induced the expression of both Foxo1 and c-Myc, thereby leading to increased levels of key transcription factors required for plasma cell differentiation (e.g., interferon regulatory factor 4 (IRF4) and Bach2) [87]. Moreover, active GSK3-β limited the CD40L-induced metabolism of GC B-cells which typically require a high glycolytic activity to sustain their growth and proliferation in the hypoxic microenvironment of GCs [88]. The glycolytic phenotype of GC B-lymphocytes is therefore stringently dependent on GSK3-β inactivation and c-Myc upregulation, as c-Myc is a well-known driver of a transcriptional program which promotes glycolysis [89]. Overall, all of these findings highlight the key roles played by GSK-3β in the physiology of GC B-cells, including those committed to plasma cell differentiation. They are also intriguing in light of the involvement of GSK3-β in B-cell NHLs which mostly derive from GCs, as we will see in this article [84]. 

## 3. GSK-3 Signaling in CML

CML is a myeloproliferative neoplasm with an incidence of 1–2 cases/100,000 adults and accounts for ~15% of newly diagnosed cases of leukemia in adults. Therefore, every year approximately 9000 new CML cases are diagnosed in the USA [90]. CML is characterized by the Philadelphia chromosome (Ph^+^), which consists of the fusion of the Abelson gene (*ABL1*) from chromosome 9q34 with the breakpoint cluster region (*BCR*) gene located on chromosome 22q11.2. This event results in the expression of an oncogenic protein referred to as BCR-ABL1 which is a constitutively active tyrosine kinase [91]. BCR-ABL1 drives the aberrant survival and proliferation of hematopoietic stem cells (HSCs) through the activation of multiple downstream pathways, including rat sarcoma (Ras)/Rapidly accelerated fibrosarcoma (Raf)/MEK/ERK, Wnt/β-catenin, Janus kinase (Jak)/Signal Transducer and Activator of Transcription (STAT), phosphatidylinositol 3-kinase (PI3K)/Akt/mechanistic target of rapamycin (mTOR), and SHH [92,93,94]. 

CML is a unique disorder, as it comprises three clinical phases: a chronic phase (CP), an accelerated phase (AP), and a blastic phase (BP). The majority (90–95%) of patients present in CP which could be asymptomatic in up to 50% of cases [90]. The CP is characterized by the expansion of myeloid cells in the bone marrow (BM), although cells are still able to differentiate and function normally [95]. CP, if it is not treated or is unresponsive to therapy, progresses to AP, characterized by the appearance of immature cells in both the BM and peripheral blood, as well as by more frequent symptoms [96]. The final stage is BP, where immature myeloid cells predominate in both BM and peripheral blood, while patient survival is measured in a few months [95].

Until 2000, CML pharmacological regimens were limited to nonspecific drugs such as busulfan, hydroxyurea, and interferon-α (IFN-α) [97], which were complemented by BMT [98]. In 2001, the therapeutic landscape of CML changed dramatically when the FDA approved imatinib mesylate, an ATP-competitive TKI that potently inhibits BCR-ABL1 enzymatic activity [99]. 

Imatinib and its second- and third-generation derivatives (nilotinib, dasatinib, bosutinib, and ponatinib) have completely changed the course and the outcome of CML, thereby improving the 10-year survival rate from ~20% to 80–90% [95]. Nevertheless, 2–3% of CML patients do not respond to TKIs and progress to AP and/or BP, with a discouraging overall survival of only 7 to 12 months [100,101]. Acquired resistance to TKIs due to several mechanisms is also a common problem, as it develops in about 25% of CML patients [102].

Moreover, it should be considered that BCR-ABL1 inhibitors cannot cure CML, as the CML leukemic stem cells (LSCs) are resistant to TKIs. This might be due to the fact that quiescent CML LSCs do not have an absolute requirement for BCR-ABL1 tyrosine kinase activity for their survival and self-renewal, although this is a controversial aspect [103,104]. Consequently, only a few patients may attempt therapy discontinuation without relapsing [105]. 

Regarding GSK-3, it was observed that the BP of CML is characterized by the aberrant activation of the Wnt/β-catenin signaling pathway in the granulocyte–macrophage progenitors (GMPs) [106]. It was subsequently discovered that CML GMPs display an in-frame splice deletion of *GSK3B* which yields a GSK-3β form which does not interact with Axin and cannot phosphorylate β-catenin. Therefore, the leukemic cells have high levels of active β-catenin accompanied by an increased serial engraftment potential in mice. In contrast, enforced expression of a full-length GSK-3β decreases β-catenin expression and reduces both the in vitro replating capability and the in vivo engraftment potential of the malignant GMPs [107]. These findings suggest that CML BP may be at least partly driven by an aberrant GSK-3β/β-catenin signaling which allows β-catenin to co-ordinate the CML LSC self-renewal. Nevertheless, it should be considered that only 13 patients in the various CML clinical phases were studied and the genetic alteration was detected in 5 specimens. Therefore, the clinical significance of this finding is somehow limited. 

However, it should be considered that other mechanisms are likely involved in the stabilization of β-catenin which is observed in CML, including the stabilizing effect of BCR-ABL1 itself on β-catenin [108]. Regarding the reason for mis-splicing of GSK-3β, it was discovered that CML transition from CP towards BP is accompanied by an increase in the adenosine deaminase acting on the RNA 1 (ADAR1) enzyme. Among other functions, ADAR1 is involved in the production of the mis-spliced form of GSK-3β [109]. Interestingly, a selective inhibitor of Proviral integration site for Moloney murine leukemia virus-1 (PIM-1) kinase (SMI-4a) decreased the levels of GSK-3β phosphorylated on Ser 9 and inhibited nuclear translocation of β-catenin in both K562 and imatinib-resistant K562 (K562/G) CML cell lines [110]. As a result of treatment with the inhibitor, both the parental and the imatinib-resistant cells underwent apoptosis which was accompanied by an upregulation of proapoptotic Bax and poly(ADP-ribose) polymerase-1 (PARP) as well as by a downregulation of antiapoptotic Bcl-2 and c-Myc. Moreover, SMI-4a negatively affected the clonogenic activity of K562 and K562/G cells [110]. These observations support the concept that the GSK-3β/β-catenin axis is also involved in the development of TKI resistance. Nevertheless, it is unclear how inhibition of PIM-1 kinase could impact GSK-3β activity.

Furthermore, pharmacological downregulation of GSK-3β by the nonselective inhibitor SB216763 reduced the cytotoxicity of ponatinib (a third-generation TKI active also against the T315I BCR-ABL1 variant) as well as the degradation of c-Myc and Mcl-1. Therefore, all of the aforementioned findings strongly indicate that GSK-3β could act as a tumor suppressor in CML, most likely via the proteasome [111].

However, there is a study hinting at a possible protumorigenic role played by GSK-3β in CML. It was demonstrated that GSK-3β is constitutively phosphorylated at Tyr 216 (hence active) and predominantly located in the cytoplasm of CD34^+^ CML LSCs, while CD34^+^ HSCs from healthy donors did express some Tyr 216 (hence active) p-GSK-3β, but also displayed Ser 9 (hence inactive) p-GSK-3β [112]. Moreover, in normal CD34^+^ cells, GSK-3β was both cytoplasmic and nuclear. Under growth-factor-rich culture conditions, imatinib increased the levels of Tyr216 p-GSK-3β and its nuclear localization. These effects were related to the formation of clusters of signaling molecules containing, besides GSK-3β, Jak2 and p60-Src kinase [113], in agreement with a previous report [114]. Interestingly, treatment with UO126 (a MEK inhibitor) or dasatinib, which inhibits both BCR-ABL1 and p60-Src kinase, decreased Tyr216 p-GSK-3β and its migration to the nucleus. This observation suggests that both MEK and p60-Src kinase are responsible for GSK-3β activation which is part of the compensatory response activated by imatinib in CML LSCs. Treatment with SB216763 led to an almost complete suppression of CML LSCs when combined with imatinib but not dasatinib, whereas it had no effects in CD34^+^ HSCs from healthy donors [112]. It was concluded that drugs targeting GSK-3β might be valuable tools for eradicating CML LSCs in combination with imatinib but not dasatinib. Nevertheless, the relevance of these observations is limited by the use of a pharmacological inhibitor that is not selective for GSK-3β (SB216763), while no genetic modulation of the expression levels of GSK-3β was performed. Therefore, a convincing demonstration of an oncogenic role played by GSK-3β in CML LSCs is still missing. 

## 4. GSK-3 Signaling in CLL

CLL is a clonal proliferation of CD5^+^/CD19^+^/CD23^+^ small B-lymphocytes which accumulate in BM, peripheral blood, and lymphoid organs [115]. CLL is a disease of the elderly (median age at diagnosis is ~72 years). It is the most common form of leukemia in adults (about 40% of all leukemias cases), with an incidence of 5.82 cases/100,000 inhabitants in the USA [116,117]. 

Chemotherapy (fludarabine and cyclophosphamide) has been the mainstay of CLL treatment for many years and is still used in combination with monoclonal antibodies targeting the CD20 antigen [118]. However, over the last 20 years, the treatment of either naïve or relapsed/refractory CLL patients has undergone dramatic changes due to the introduction of targeted therapeutics which include the Bruton tyrosine kinase (BTK) inhibitor ibrutinib [119] and the Bcl-2 inhibitor venotoclax [120], and the PI3K inhibitors idelalisib [121] and duvelisib [73]. These drugs are used either as monotherapy or in combination with the glycoengineered monoclonal antibody obinutuzumab [122]. Although the outcome of CLL patients is improved by all of these novel therapeutics, especially in high-risk disease [123], adaptive resistance to targeted therapy inevitably occurs, thereby leading to leukemia progression [124]. Therefore, there is still room for advancement in the field of CLL-targeted drugs. 

CLL is a highly heterogeneous disorder characterized by several abnormalities affecting genes related to response to DNA damage and cell cycle control, RNA processing, and cytokine signaling. These mutations impact both the pathobiology and the outcome of the disease by altering several signal transduction networks, including the Wnt/β-catenin pathway [125]. Earlier studies demonstrated that CLL B-cells, when compared with healthy B cells, displayed higher expression levels of several Wnt family proteins (Wnt3, Wnt5b, Wnt6, Wnt10a, Wnt14, and Wnt16) and of their cognate Frizzled (Fzd) receptor, Fzd3 [126]. Moreover, the neoplastic B-cells exhibited upregulation of the Wnt/β-catenin-regulated transcription factor Lymphoid-enhancing factor-1 (LEF-1) and of its downstream target, cyclin D1. Furthermore, the GSK-3 inhibitor SB216763 diminished the levels of GSK-3β phosphorylated on Tyr216, thereby increasing the survival of CLL B-lymphocytes [126]. More recently, however, the existence of an extrinsic mechanism which leads to upregulation of β-catenin levels has been demonstrated [127]. Specifically, it was shown that CLL cells induce Notch 2 activity in BM mesenchymal stromal cells (MSCs), which is required for the transcription of the complement factor, C1q. In turn, C1q derived from MSCs somehow inhibits GSK3-β-mediated degradation of β-catenin, thereby contributing to the activation of Wnt/β-catenin signaling in CLL cells [127]. Therefore, these two studies suggest that GSK-3β could act as a tumor suppressor in CLL cells. However, GSK-3β could also display a tumor-enhancing role, as it has been reported that it promotes NF-κB binding to target genes (e.g., *XIAP*, *BCL2*), given that in CLL cells GSK-3β accumulates in the nucleus [128]. In the latter case, GSK-3 inhibition by AR-A014418 enhanced apoptosis in CLL B-cells via downregulation of the expression of X-linked inhibitor of apoptosis protein (XIAP) and Bcl-2. Overall, CLL is a neoplastic disease where GSK-3β seems to play two contrasting roles. 

## 5. GSK-3 Signaling in MM

Multiple myeloma (MM) is an aggressive disease characterized by the clonal expansion of transformed plasma cells within the BM. The incidence of MM has been steadily increasing over the last three decades and MM represents nearly 10–13% of all hematological cancers, being the second most frequent blood malignancy [129]. MM frequency augments with age and peaks during the sixth and seventh decades of life, with 32,000 new cases diagnosed in the USA and approximately 13,000 deaths [130]. A retrospective analysis on the global burden of MM in 2006–2016 published in 2018 reported an incidence rate of 2.1 cases per 100,000 inhabitants [130]. The 5-year survival rate of MM patients is ~55%. Nevertheless, due to continuing advances in therapeutic regimens based on proteasome inhibitors and immunomodulatory drugs [131], the survival rates are improving [130]. 

A number of studies have investigated the expression and function of GSK-3 in MM cells. Initial investigations showed that GSK-3 inhibitors induced apoptosis in MM cell lines, dephosphorylated forkhead transcription factors FoxO1 and FoxO3a, and activated the cyclin dependent kinase inhibitor, p27^kip1^ [132]. 

A more detailed analysis demonstrated that both the GSK-3 paralogs were abundantly expressed in MM. However, in some MM patient primary cells and in all the MM cell lines, GSK-3β expression levels were lower than those of GSK-3α. Interestingly, GSK-3β was more abundantly phosphorylated on Ser9 than GSK-3α on Ser21 in both normal B cells and MM cells, whereas GSK-3α was fairly more abundantly phosphorylated on Tyr279 than GSK-3β on Tyr216 [133]. These observations might indicate that GSK-3α is the predominating active isoform in malignant plasma cells. Two different pharmacological inhibitors (SB216763 and SB415286) caused MM cell proliferation arrest and apoptosis [133]. These findings must be interpreted with caution, as the drugs are not GSK-3 selective. Indeed, they also inhibit cyclin-dependent kinases (CDK)s which share 33% amino acid identity with GSK-3 [134,135]. In this connection, it is worth remembering that the CDK inhibitor AT7519 induced apoptosis in MM cells, which was accompanied by dephosphorylation of GSK-3β on Ser9 and an increased phosphorylation of the GSK-3β substrate, glycogen synthase [136].

Under basal conditions, a decrease in MM cell viability was also observed upon siRNA-directed downregulation of GSK-3β, but not of GSK-3α [133]. The genetic downmodulation of GSK-3 isoforms also differentially affected MM cell sensitivity to the cytotoxic effects of the proteasome inhibitor, bortezomib. Bortezomib alone induced a marked reduction in Ser 21/9 p-GSK-3 levels as well as an increase in Tyr279/216 p-GSK-3 levels. Therefore, bortezomib caused an increase in GSK-3 activity that, quite surprisingly, could be counteracted only by genetic downmodulation of GSK-3α [133]. Overall, these findings seem to indicate that the two paralogs may have similar, but not overlapping, effects on MM cell survival. 

**Figure 4:**
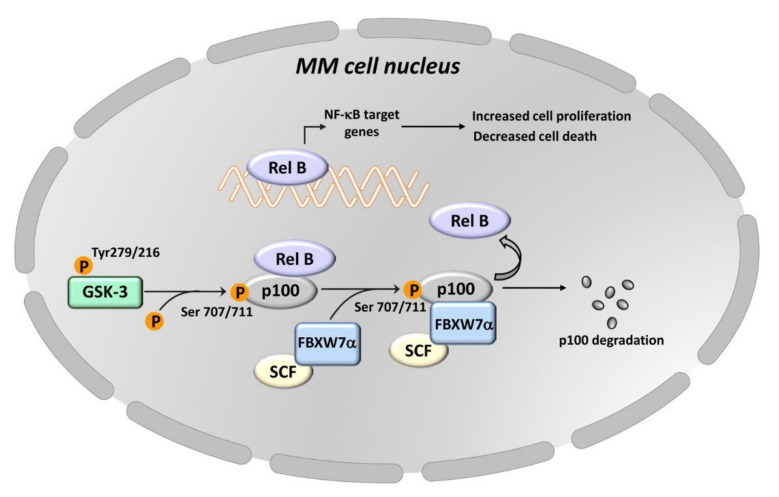
GSK-3 promotes the proliferation and survival of MM cells via the noncanonical NF-κB pathway. GSK-3 phosphorylates the NF-κB p100 inhibitor on Ser707/711 residues, thereby allowing the binding of the FBXW7α/SCF complex to p100. p100 is then degraded through the proteasome. As a consequence, the transcription factor RelB is free to enhance the transcription of genes fundamental for cell proliferation and survival. The GSK-3 isoform involved in p100 degradation in MM cells has not been identified yet.

GSK-3 could also promote survival of MM cells through the noncanonical NF-κB pathway. It is worth remembering here that this signaling pathway is involved in regulating different aspects of immune functions and tumorigenesis [137,138]. In some MM cell lines and primary samples, the noncanonical NF-κB pathway is overactive via the interactions between FBXW7α (i.e., the nuclear isoform of FBXW7) and GSK-3 [139]. The target of these interactions is p100, i.e., the physiological inhibitor of the noncanonical NF-κB pathway [140]. GSK-3 phosphorylates p100 on Ser707/711, thereby allowing the binding of the SCF/FBXW7α complex to p100 which is then degraded via the proteasome [139]. It is not clear where p100 degradation occurs; however, the proteasome is present also in the nucleus [141]. Whatever the case, once freed from its interactions with p100, RelB is able to bind at the promoters of NF-κB target genes which are upregulated [139] (Figure 4). Pharmacological inhibition of GSK-3 activity led to MM cells apoptosis, which was at least partially dependent on the increased levels of p100 in the cell nucleus and downregulation of NF-κB activity [139]. The GSK-3 paralog which phosphorylates p100 is unknown, as the authors used drugs that are not isoform-selective (6-bromoindirubin 3′-oxime (BIO), CHIR99021, and compound A) to inhibit GSK-3. All of these compounds target both GSK-3α and GSK-3β with equal potency [134,142,143].

Another critical target of GSK-3 in MM is represented by the musculoaponeurotic fibrosarcoma oncogene homolog (Maf) family of transcription factors. This family comprises four members, MafA, MafB, c-Maf, and neural retina-specific leucine zipper protein (NRL) [144]. Maf transcription factors play important roles in the differentiation of several types of cells, including pancreatic β-cells, kidney podocytes, osteoblasts, and macrophages. Moreover, they are involved in several human diseases, such as congenital renal, eye, and bone disorders, as well as diabetes and cancer [144]. In MM, three genes encoding for Maf transcription factors are target partners of the IgH locus in chromosomal translocations found in neoplastic plasma cells: *c-MAF* in the t(14;16) translocation; *MAFB* in the t(14;20); and *MAFA* in the t(8;14) [145]. Although they are rare events, these translocations are associated with high Maf protein expression levels and with a poor overall survival in MM patients [146]. The increased levels of Maf proteins play an important role in MM pathogenesis, through the regulation of cyclin D2, integrin β7, CCR1, ARK5, and DEP domain-containing mTOR-interacting protein (DEPTOR) expression [147,148,149,150,151].

GSK-3 sequentially phosphorylates MafA protein on Ser 61, Thr57, Thr53, and Ser49 residues in rat pancreatic β cells, INS-1. These phosphorylations reduce the half-life of the transcription factor as they accelerate ubiquitin-dependent proteasomal degradation. However, they also increase the transactivation activity via the recruitment of the P/CAF coactivator (also known as histone acetyltransferase 2B) which in turn protects MafA from ubiquitination and proteasomal degradation [152]. Interestingly, genetic downmodulation by shRNAs demonstrated that depletion of both GSK-3α and GSK-3β strongly reduced MafA phosphorylation, whereas depletion of just one paralog was insufficient. This finding suggests that both GSK-3 isotypes phosphorylate MafA [152]. MafB and c-Maf are phosphorylated by GSK-3 also in MM [146,153,154]. Treatment of MM cells with nonselective GSK-3 inhibitors (LiCl, SB216763) decreased the phosphorylation levels of Maf proteins and increased their expression via a downregulation of their degradation, as expected [146,153,154]. DEPTOR expression was decreased in response to LiCl treatment in MafB- and c-Maf-expressing MM cells, as expected. Moreover, LiCl downmodulated both proliferation and colony formation of MAF-expressing MM cell lines [146]. These findings seem to indicate that the maintenance of the phosphorylation status is essential to preserve the MabB- and c-MAF-transforming activity in MM. Therefore, this study suggests that patients displaying an MAF-driven MM may benefit from therapies targeting GSK-3. Regarding the use of LiCl, it should be pointed out this drug has a dual manner of inhibiting GSK-3, by a direct inhibition with a high IC_50_ but also by an indirect manner via an increase in the Ser-phosphorylated GSK-3 forms. Lithium salts compete with Mg^2+^; hence, they inhibit other enzymes that are Mg^2+^-dependent, including some phosphatases [155,156]. It might be that the LiCl effects on Ser-phosphorylated GSK-3 forms are somehow related to phosphatase inhibition. 

Whatever the case, it should be pointed out that MAF proteins mediate intrinsic resistance to proteasome inhibitors [153,154]; hence, an increase in their expression due to GSK-3 inhibition might aggravate refractoriness to this class of drugs. Whatever the case, the relevance of all of these findings is limited by the use of first-generation GSK-3 inhibitors and by the lack of a clear understanding of the isoform(s) involved in MAF protein phosphorylation in MM cells. 

There is a study which demonstrates how GSK-3 potentially has tumor-suppressive functions in MM; however, its activity is restrained by Akt activation, thereby resulting in the stabilization of Mcl-1 levels [157]. Another restrainer of GSK-3 in MM cells is the histone demethylase KDM4C, which is upregulated in MM patients and, when overexpressed in MM cell lines, increases β-catenin levels and activity while decreasing both the RNA and protein expression of GSK-3β [158]. Finally, there is evidence that lenalidomide (a drug widely used for treating MM patients [159]) induces activation of Wnt/β-catenin signaling. The authors related the effects of lenalidomide in part to the suppression of casein kinase 1α expression and in part to the phosphorylation of GSK3α/β on serine residues [160]. Nevertheless, as we have discussed in Section 2, inhibitory phosphorylation of GSK-3 on Ser 21/9 residues is unlikely to be the driving force which unleashes β-catenin migration to the nucleus. 

In general, quite a few of the studies quoted in this Section demonstrate that GSK-3 could act as a prosurvival factor in MM cells. Nevertheless, there are findings suggesting that GSK-3 could also display prodeath activity. However, the data are largely incomplete and mostly based on the use of nonselective pharmacological inhibitors. Therefore, a more detailed analysis of the roles played by GSK-3 paralogs and of their relevance as therapeutic targets in the setting of MM is required before any firm conclusions can be drawn.

## 6. GSK-3 Signaling in B-Cell NHLs

B-cell NHLs are the most frequent hematologic malignant disorders, being among the top 10 most frequent neoplasia worldwide, and are by far much more common than T-cell-derived NHLs [161]. The 2016 World Health Organization (WHO) classification of B-NHL has recognized approximately 60 distinct entities [2]. The two most frequent subtypes of B-cell NHL are diffuse large B-cell lymphoma (DLBCL), which accounts for 35% of all B-cell NHLs, and follicular lymphoma (FL), which accounts for up to 20–25% of B-cell NHLs. Interestingly, these two subtypes originate from GC lymphoid cells [88], although some DLBCLs are from a post-GC origin, and are referred to as “activated B-cell” (ABC)-type DLBCL [162]. Another subtype of aggressive B-cell NHL arising from GC lymphoid cells is Burkitt lymphoma, whose endemic variant is almost invariably associated with Epstein–Barr virus (EBV) infection. In contrast, the sporadic variant of Burkitt lymphoma is rarely associated with the EBV [163]. Interestingly, the aforementioned subtypes of B-NHLs are characterized by a dysregulation of c-Myc signaling [164]. One of the most important signaling molecules for malignant B-cell proliferation, survival, and drug-resistance is the BCR whose activity is aberrantly regulated in many patients with B-cell NHLs due to multiple reasons [165]. For example, in the DLBCL subtype mutations in the *CARD11*, *MYD88*, and *CD79A/B* genes contribute to the perpetuation of the signals downstream of the BCR, thereby enhancing proliferation and survival of the neoplastic B-cells [166]. Therefore, the BCR signaling network has become an attractive target in the therapy of B-cell NHLs. 

**Figure 5:**
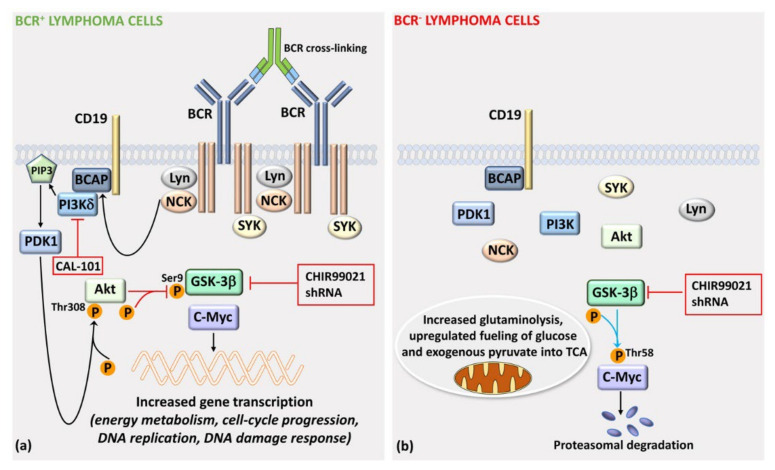
BCR-mediated inactivation of GSK-3β upregulates c-Myc-dependent gene transcription in mouse and human B-cell lymphoma. (**a**) Upon BCR cross-linking (which mimics aberrant BCR upregulation), a PI3Kδ/PDK1/Akt axis leads to inhibitory phosphorylation of GSK-3β. As a consequence, c-Myc is not degraded via the proteasome and increases the transcription of a set of genes involved in metabolism, cell cycle progression, and DNA replication and repair. The PI3Kδ CAL-101 could oppose c-Myc-dependent transcription. The GSK-3β inhibitor CHIR99021 or shRNA decreased transcription of C-Myc-regulated genes. (**b**) In cells where the BCR has been genetically depleted, GSK-3β is active; hence, it phosphorylates c-Myc on the Thr58 residue, thereby leading to its proteasomal degradation. The GSK-3β inhibitor CHIR99021 or shRNA downregulates c-Myc degradation. The BCR- cells try to compensate their metabolic vulnerability via increased glutaminolysis and upregulated fueling of glucose and exogenous pyruvate into TCA. Abbreviations used: SYK, spleen tyrosine kinase; Lyn, Lck/Yes novel tyrosine kinase; NCK, noncatalytic region of tyrosine kinase; BCAP, B-cell adaptor for PI3K; PIP3, phosphatidylinositol (3,4,5) trisphosphate.

Recent evidence has highlighted the involvement of the BCR signaling in mitigating GSK-3 activity in some B-cell NHLs. It was observed that, in c-Myc-driven mouse B-cell lymphomas and human Burkitt lymphoma cells, genetic ablation of BCR does not, per se, preclude the growth of neoplastic cells. However, the ablation leads to a disadvantage in the competitive growth in vitro and in vivo with BCR^+^ cells in which BCR had been cross-linked and activated [167]. In particular, receptorless B-lymphoma cells show a significantly delayed G_1_/S transition and increased apoptosis in comparison with their BCR^+^ counterparts. The BCR^-^ lymphoma cells display a rewiring of their metabolism, consisting in upregulated glutaminolysis and increased fueling of both glucose and exogenous pyruvate into the tricarboxylic acid (TCA) cycle. Nevertheless, they are more sensitive to aminoacid starvation than BCR^+^ cells. As expected, BCR^-^ lymphoma cells show decreased levels of GSK-3β phosphorylated on Ser9 through PI3Kδ/phosphoinositide-dependent kinase 1 (PDK1)/Akt signaling (Figure 5). Either GSK-3β genetic knockdown or chemical inhibition by CHIR99021 rescues the proliferative capability of BCR^-^ lymphoma cells to levels similar to those of BCR^+^ cells [167]. More interestingly, the expression of approximately 50% of BCR-dependent genes is modulated via c-Myc through a PI3Kδ/PDK1/Akt/GSK-3β axis. These genes are related, among other functions, to energy metabolism, cell-cycle progression, DNA replication, and DNA damage response [167] (Figure 5). Overall, these findings demonstrate that when GSK-3β activity is restrained through the BCR/PI3Kδ/PDK1/Akt axis, the optimal fitness of lymphoma cells is bolstered via c-Myc, a phenomenon which is reminiscent of the mechanisms controlling proliferation and survival of more mature healthy B-cells in GCs (see Section 2.1). However, in B-cell NHLs, GSK-3β activity could be also downmodulated via proteasomal degradation of GSK-3β itself, due to interactions with Inhibitor of Bruton’s tyrosine kinase α (IBTKα). As a consequence of GSK-3β proteolysis, the protein levels of β-catenin increase, thereby resulting in the transcriptional activation of the *MYC* and *CCND1* target genes [168].

Nevertheless, there is also evidence that GSK-3 inhibition with the clinically relevant GSK-3 inhibitor 9-ING-41 could decrease proliferation and increase apoptosis in DLBCL cell lines and patient primary samples [169,170]. More importantly, either deletion of the GSK-3β gene through CRISPR-Cas9 technology or GSK-3β knockdown by shRNA leads to lymphoma cell growth arrest at the G_2_/M phase of the cell cycle [170]. Interestingly, GSK-3β, but not GSK-3α, was found to be essential for cell progression through the prophase stage of mitosis. However, the molecular mechanisms underlying this effect are still awaiting clarification, although GSK-3β has been shown to bind to centrosomes and mitotic spindles in lymphoma cells [170]. These findings, along with those from other investigators [171], have paved the groundwork for a phase 1 trial of 9-ING-41 (NCT03678883), which is currently underway [172].

Inhibition of GSK-3 has been also claimed as a modality to increase c-Myc-driven apoptosis of chemo-resistant Burkitt lymphoma cells, as GSK-3 inhibitors upregulate c-Myc protein half-life via a paradoxical transient attenuation of its proteasomal degradation [173]. Nevertheless, the exact mechanisms leading to increased chemo-sensitivity are far from being completely understood, although they could be dependent on altered gene expression.

In general, the findings summarized in this section show once again the dual role played by GSK-3 in cancer settings. 

## 7. Role of GSK-3 in the Immunosuppressive Microenvironment of Chronic Hematological Malignancies

Several lines of evidence demonstrate that GSK-3 isoforms, and especially GSK-3β, are mediators of anticancer immune response [174,175]. The immunomodulatory functions of GSK-3β occur both in vitro and in vivo. GSK-3β appears to upregulate the expression of immune checkpoint molecules through the transcriptional activation of Programmed cell death 1 (PD-1) in T-cells [176]. Accordingly, inhibition of GSK-3β activity using siRNA technology or a pharmacological inhibitor (SB415286) led to reduced levels of PD-1 and enhanced the activity of cytotoxic T-cells [177]. The reduction of PD-1 levels and the enhancement of cytotoxic T-cell activity have been detected in the context of various syngeneic murine cancer models, including pancreatic carcinoma [178]. Moreover, the inhibition of GSK-3β has been shown to complement the functions of the costimulatory molecule, CD28, in the proliferation of human T-cells [179] and has been demonstrated to increase the survival and enhance the anticancer cytotoxicity of CAR T-cells in a glioblastoma preclinical setting [180]. Furthermore, GSK-3β inhibition by CHIR99021 enhances the maturation, expansion, and antibody-dependent cytotoxicity of natural killer (NK) cells. Mechanistically, treatment with CHIR99021 leads to an increased production of proinflammatory cytokines (IFN-γ, tumor necrosis factor α (TNFα)) and upregulates NK cytotoxicity against ovarian cancer cells [181]. Therefore, GSK-3β inhibition has the potential to directly reinforce the immunoreactivity of T- and NK cells infiltrating the tumor microenvironment.

Regarding chronic hematological malignancies, recent findings have shown that GSK-3α decreases NK cells reactivity against imatinib-resistant CML cells as the paralog diminishes the expression of NK group 2 member D (NKG2D) and NK protein 30 (NKp30) [182]. NKG2D and NKp30 are among the most important NK cell-activating receptors [183]. Interestingly, GSK-3α inhibition enhances leukemic cell susceptibility to NK cell cytotoxicity both in vitro and in vivo [182]. However, there also is genetic evidence that GSK-3β synthesized by CML cells might be involved in TKI resistance evoked by IFN-γ secreted by microenvironmental T- and NK cells [184]. Accordingly, CML cells where GSK-3β had been knocked down displayed higher levels of apoptosis relative to control cells upon treatment with IFN-γ [185]. These findings revealed additional mechanisms leading to the development of TKI resistance in CML cells.

In MM, cellular inhibition of GSK-3 via nonselective inhibitors (LiCl, SB216763, or BIO) increased the surface expression of the NKG2D ligand, MHC class I polypeptide–related sequence A (MICA), thereby leading to enhanced recognition and killing of neoplastic plasma cells by NK cells [186]. The effects on MICA expression were independent from β-catenin and could be related to enhanced activity of its promoter, which is usually repressed under basal conditions through active STAT3 signaling. STAT3-dependent repression of the MICA gene could be released by GSK-3 inhibitors which decreased the constitutive levels of p-Tyr705 STAT3 [186]. This observation suggests that in MM cells, phosphorylation of STAT3 on Tyr705 is dependent on GSK-3 activity. Although GSK-3 is mainly known for phosphorylating STAT3 on Ser/Thr residues [187], it has the capacity of phosphorylating STAT3 also on Ty705, either indirectly via membrane-associated tyrosine kinases [188] or directly [189]. 

As far as B-cell lymphomas are concerned, inactivation of GSK-3α/β during priming could substitute CD28 costimulation in potentiating cytotoxic T-cell functions against murine EL4 target lymphoma cells [176].

Therefore, the findings summarized in this section suggest that downmodulation of GSK-3 activity may result in improved immunoreactivity of both T- and NK cells infiltrating the microenvironment of chronic hematological cancers.

## 8. New Strategies for Targeting GSK-3 in Cancer Cells

The development of potent, isoform-specific GSK-3 inhibitors has proven challenging. Indeed, the amino acid sequence around the ATP-binding pocket of GSK-3 paralogs is nearly identical [190]. Therefore, the majority of inhibitors which have been synthesized compete with the ATP-binding site of GSK-3 paralogs with low selectivity. Nevertheless, isoform-selective inhibitors have been disclosed, including BRD0705, compound 27, and compound 28_14 (which target GSK-3α [82,191,192]) or TWS199 (which targets GSK-3β [193]). Both BRD0705 and compound **28**-**14** have been tested in preclinical models of acute myelogenous leukemia (AML) with promising results [194]. Obviously, it would be interesting to test these selective inhibitors also in preclinical models of chronic hematological malignancies. 

Over the last ten years, much interest has surrounded the development of irreversible inhibitors as anticancer therapeutics due to their capacity of forming covalent bonds with target proteins rather than binding via noncovalent interactions, as most of the conventional kinase inhibitors do [195]. Several covalent inhibitors targeting kinases critical for cancer pathobiology (EGFR, JAK3, BTK, FAK, p60 Src, etc.) have been disclosed, and in 2019, K-RasG12C covalent inhibitors have entered clinical trials [196]. Covalent inhibitors display at least three advantages over conventional inhibitors. First, they usually have a binding affinity for their targets higher than conventional therapeutics; hence, they are capable of also targeting shallow binding sites. Second, they potentially have an extended duration of action which could allow less-frequent administration. Third, they could specifically target only one out of a group of closely related kinases [197]. However, irreversible covalent inhibitors have the potential obvious disadvantage of becoming permanently bound to their targets in healthy cells, thereby causing severe adverse effects. To overcome this limitation, some investigators have synthesized reversible covalent compounds which target the SH group of noncatalytic cysteine residues in kinases [198]. Nevertheless, cysteine residues are rare in the human kinome [199]. A solution to this issue would be to target lysine residues, as they are frequently found in the human proteome, quite often at the protein active sites [200]. A reversible inhibitor targeting lysine residues has been disclosed very recently [201]. Regarding GSK-3, there are some examples of irreversible covalent inhibitors targeting cysteine residues [202,203,204]. Among these drugs, compound **4**-**3** seems particularly interesting, as it targets the unique Cys14 residue found in GSK-3β and inhibits cell growth in an acute promyelocytic leukemia murine model [204].

An additional class of drugs which may be worth testing in chronic malignant hematologic malignancies is represented by allosteric GSK-3 inhibitors, as they display enhanced selectivity, thereby reducing the chance of producing adverse effects [205,206]. 

Another novel technology that could be employed for selectively eliminating the activity of the GSK-3 paralogs in neoplastic cells is based upon the concept of proteolysis targeting chimera (PROTAC). PROTACs are heterobifunctional therapeutics which simultaneously bind a target protein and an E3 ubiquitin ligase, thereby enabling the selective ubiquitination and proteasomal degradation of their targets [131]. An advantage of PROTACs is that they can degrade proteins previously thought to be undruggable (i.e., transcription factors). Another benefit of using this technology is that one PROTAC molecule is capable of inducing multiple rounds of degradation; hence, therapy requires less drug exposure. Therefore, in general, PROTACs display a lower toxicity in comparison to conventional inhibitors, although there are still unresolved issues including a relatively high molecular weight and potential off-target activity [207]. Third generation, light switchable PROTACs are now being developed to limit the problem of uncontrolled protein degradation in any cells in an organism [208]. 

Very recently, the first PROTAC selectively acting on GSK-3β (referred to as PG21) has been disclosed [58]. PT-65 is another PROTAC targeting GSK-3β. Interestingly, PT-65 was capable of attenuating GSK-3β-mediated tau hyperphosphorylation, thereby alleviating the amyloid-β (Aβ) peptide-induced SH-SY5Y cell damage and ameliorating learning and memory impairments in a rat model of Alzheimer’s disease [209]. Therefore, these PROTAC molecules might also be effective therapeutics in cancer settings where overactive GSK-3β plays a tumor-promoter role. 

Besides PROTACs, nanoparticle-based drug delivery systems have the potential for improving the current anticancer therapies, as they offer several advantages including enhanced solubility, bioavailability, and stability of the carried drugs [210]. Nanocarriers could enhance the efficacy of drugs by optimizing their biochemical and pharmacokinetic characteristics. Therefore, patients might tolerate higher doses of drugs while experiencing less serious adverse effects [211,212]. Nanocarriers could also be employed for the purpose of specifically targeting drugs to an organ or even cells of interest, as exemplified by the use a bone-targeted nanoparticle for delivering of a GSK-3β inhibitor for the treatment of bone fractures [213]. Other examples of nanocarrier-based drug delivery systems targeting GSK-3 are available in the literature [178,214,215]. One of the existing platforms was developed for the delivery of a silicasome-encapsulated GSK-3α/β inhibitor (AZD1080) to improve immunotherapy in syngeneic murine models of colorectal, pancreatic, and lung cancers. The therapeutic effectiveness of the encapsulated drug was similar or even better than an anti-PD-1 antibody; however, the treatment was devoid of toxicity. Interestingly, free AZD1080 displayed no significant effects on cancer growth inhibition [178]. Therefore, the efficacy of nanocarriers delivering GSK-3-targeted drugs certainly warrants further investigation in chronic malignant blood diseases [216].

Overall, our arsenal of drugs targeting GSK-3 will certainly be increasing over the next few years, and novel therapeutics may lead to significantly better achievements also in cancer settings. 

## 9. Perspectives and Conclusions

GSK-3 is a multitasking kinase located at the crossroad of numerous signaling pathways critical for many aspects of cancer cell pathobiology. This finding has brought GSK-3 to the attention of both the academy and pharmaceutical companies. However, the development of efficacious drugs targeting GSK-3 has proven to be a difficult task, due to several reasons. These include: the high similarity in the ATP-binding sites of the two paralogs; the many GSK-3 substrates whose targeting might disarray functions of vital importance in healthy cells; the partial redundancy of the two isoforms; and the opposing roles played by GSK-3 in the same disorder. Therefore, although several GSK-3 inhibitors have been evaluated in preclinical studies, relatively few have reached phase 2 clinical trials [174,217]. In particular, the nearly ubiquitous expression of GSK-3 isoforms in human tissues and organs has resulted in quite serious adverse effects, thereby leading to the failure of many compounds [217]. In a few cases, the safety profile of the inhibitor was acceptable; however, no clinical activity was observed, presumably due to the low efficacy of the drug [218]. Therefore, none of the GSK-3 inhibitors have been approved for clinical use. 

As we have summarized in this review, we have now at our disposal several novel therapeutics and platforms targeting GSK-3 which may circumvent some of the aforementioned issues. While acknowledging the relevance of these developments, much is left to be uncovered for translating them in the clinic. We need to achieve a much better understanding of the complex molecular interactions involving GSK-3 in both healthy and cancerous cells as well as the best ways to employ GSK-3 inhibitors in chronic hematological disorders. As we learn more about GSK-3 roles in individual disorders, it may be possible to develop drugs which principally target the actions of GSK-3 which are involved in pathobiology. Moreover, biomarkers indicating which patients may benefit the most from therapeutics targeting GSK-3 are still awaiting definitive identification. 

Regarding the immunosuppressive microenvironment of chronic malignant blood disorders, the relevance of GSK-3 has been studied in T- and NK cells. However, in MM patients, tumor-associated macrophages (TAMs) are a major cell subset within the tumor sites, where they could support chemoresistance, cancer cell proliferation and survival, as well as immunosuppression [219]. There are no studies on the possible roles played by GSK-3 in this context. However, a GSK-3 involvement could not be ruled out, as GSK-3 plays an important role in macrophage physiology [220], while the Wnt/β-catenin pathway has been demonstrated to be aberrantly active in TAMs in other cancer types [221]. Therefore, this is a research field that needs to be thoroughly investigated. 

In conclusion, although several significant issues still remain to be solved, all the advances made over the last few years have the potential to provide a venue for a significant scientific innovation, thereby heralding the dawn of a new era for GSK-3 inhibitor use in chronic malignant blood disorders. 

## Data Availability

Data sharing not applicable. No new data were created or analyzed in this work.
